# Evaluation of the Effects of Diet-Induced Obesity in Zebrafish (*Danio rerio*): A Comparative Study

**DOI:** 10.3390/nu16193398

**Published:** 2024-10-07

**Authors:** Maria Gabriela F. R. Silva, Ana Carolina Luchiari, Isaiane Medeiros, Augusto M. de Souza, Alexandre C. Serquiz, Fabiane F. Martins, Sérgio A. B. de Moura, Christina S. Camillo, Silvia Regina B. de Medeiros, Tatiana dos S. Pais, Thaís S. Passos, Denise M. L. Galeno, Ana Heloneida de A. Morais

**Affiliations:** 1 Nutrition Postgraduate Program, Center of Health Sciences, Federal University of Rio Grande do Norte, Natal 59078-970, RN, Brazil; magabi_rocha@yahoo.com.br (M.G.F.R.S.); thais.passos@ufrn.br (T.S.P.); 2 Psychobiology Postgraduate Program, Center of Biosciences, Federal University of Rio Grande do Norte, Natal 59078-970, RN, Brazil; ana.luchiari@ufrn.br; 3 Biochemistry and Molecular Biology Postgraduate Program, Center of Biosciences, Federal University of Rio Grande do Norte, Natal 59078-970, RN, Brazil; isaianemedeirosss@gmail.com (I.M.); silvia.batistuzzo@ufrn.br (S.R.B.d.M.); tatyh.pais@gmail.com (T.d.S.P.); 4 Biotechnology Program—Northeast Biotechnology Network (RENORBIO), Technology Center, Federal University of Rio Grande do Norte, Natal 59078-970, RN, Brazil; augustomonteiro25@gmail.com; 5 Department of Nutrition, Federal University of Paraíba, João Pessoa 58051-900, PB, Brazil; alexandreserquiz@gmail.com; 6 Department of Morphology, Center of Biosciences, Federal University of Rio Grande do Norte, Natal 59078-970, RN, Brazil; fabiane.martins@ufrn.br (F.F.M.); sergio.moura@ufrn.br (S.A.B.d.M.); christina.camillo@ufrn.br (C.S.C.); 7 Department of Cell Biology and Genetics, Center of Biosciences, Federal University of Rio Grande do Norte, Natal 59078-970, RN, Brazil; 8 Department of Nutrition, Center of Health Sciences, Federal University of Rio Grande do Norte, Natal 59078-970, RN, Brazil; 9 Multicenter Postgraduate Program in Physiological Sciences, Federal University of Rio Grande do Norte, Natal 59078-970, RN, Brazil; denise.galeno@ufrn.br

**Keywords:** high-fat diet, weight gain, inflammation, anxiety

## Abstract

Objectives: This study aimed to compare diet-induced obesity (DIO) models in zebrafish and investigate the complications and differences between sexes in biochemical and inflammatory parameters. Methods: Adult animals of both sexes were divided into four groups (*n* = 50) and fed for eight weeks: control group 1: *Artemia* sp. (15–30 mg/day/fish); control group 2: commercial fish food (3.5% of average weight); obesity group 1: pasteurized egg yolk powder + soybean oil (5% of average weight); obesity group 2: *Artemia* sp. (60–120 mg/day/fish). Dietary intake, caloric intake and efficiency, body mass index, biochemical, inflammatory, behavioral, histopathological, and stereological parameters, and inflammation-related gene expression were investigated. Results: Obesity group 1 was the most indicated to investigate changes in the anxious behavioral profile (*p* < 0.05), triglyceride elevation [52.67 (1.2) mg/dL], adipocyte hypertrophy [67.8 (18.1) µm^2^; *p* = 0.0004], and intestinal inflammation. Obesity group 2 was interesting to investigate in terms of weight gain [167 mg; *p* < 0.0001), changes in fasting glucose [48.33 (4.14) mg/dL; *p* = 0.003), and inflammatory parameters [IL-6: 4.24 (0.18) pg/mL; *p* = 0.0015]. Conclusions: Furthermore, both DIO models evaluated in the present study were effective in investigating hepatic steatosis. The data also highlighted that sex influences inflammatory changes and fasting blood glucose levels, which were higher in males (*p* > 0.05). The results show new metabolic routes to be explored in relation to DIO in zebrafish.

## 1. Introduction

Obesity is one of the most severe health problems in existence. This disease is associated with the cause of death of 2.8 million people per year worldwide. The estimate for 2025 is that 2.3 billion people in the world will be overweight, with 700 million classified as obese [1,2]. One of the causes of obesity is the consumption of diets with excess macronutrients. A diet rich in lipids (high-fat diet) impairs nutrient absorption, increases adiposity, generates low-grade chronic inflammation and insulin resistance, and can cause obesity, cardiovascular disease, and cancer development [1,2,3,4]. Furthermore, an increased intake of carbohydrates (high-carbohydrate diet) increases adipose tissue, weight gain, and hepatic steatosis. In addition, it causes changes in biochemical parameters related to various diseases, such as type 2 diabetes mellitus [5,6].

In this sense, clinical studies related to the induction of obesity in humans aimed at investigating cellular mechanisms and complications related to diets are impractical. Therefore, experimental (preclinical) models are necessary for developing such studies, and they have demonstrated legitimate results comparable to clinical trials [7,8,9].

Zebrafish (*Danio rerio*) provide an excellent low-cost, easy to maintain and handle animal model. Furthermore, this preclinical model has 70% genetic homology with humans and shares 82% genetic homology in genes associated with human diseases [10,11]. Therefore, it can be used in behavioral, genetic, and toxicological investigations and to aid in the understanding of cellular mechanisms of several human diseases. In addition, it is an excellent option for testing therapeutic agents [12,13]. Recently, studies with zebrafish have also shown advantages related to research on obesity, metabolic syndrome, and type 2 diabetes mellitus [9,12,13,14].

Diet-induced obesity (DIO) in zebrafish can generate symptoms and complications similar to those in humans [9,12,13,14]. Therefore, several studies using the above-mentioned animal model have been carried out to expand knowledge about obesity [9,12,13,14]. In this sense, it is crucial to investigate the models of induction and development of this disease, and it is of fundamental importance to develop standardized protocols that cause the expected effects.

Thus, this study was able to evaluate the effects of different models of obesity-inducing diets and develop a protocol considering the differences between the sexes in biochemical and inflammatory parameters, in addition to the specificities of the diets used for studies on the development of obesity and related diseases. This comparative study may enable investigations of the various molecules and substances with potential anti-obesity effects.

## 2. Materials and Methods

### 2.1. Animal Model

Two hundred animals of both sexes of the species *Danio rerio* (wild type, three months old, and weighing an average of 88 mg) were kept at 28 ± 1 °C, with a light/dark cycle of 14 h:10 h, at the FishLab of the Federal University of Rio Grande do Norte (UFRN). According to Westerfield et al. [15], water conditions of environmental quality were maintained. All experimental protocols followed the legislation for animal experimentation established by the National Council for Animal Experimentation of Brazil. The Ethics Committee for the Use of Animals of the Federal University of Rio Grande do Norte approved the project with protocol number 035/2022 and certificate number 301.035/2022.

### 2.2. Experimental Design

**Figure 1:**
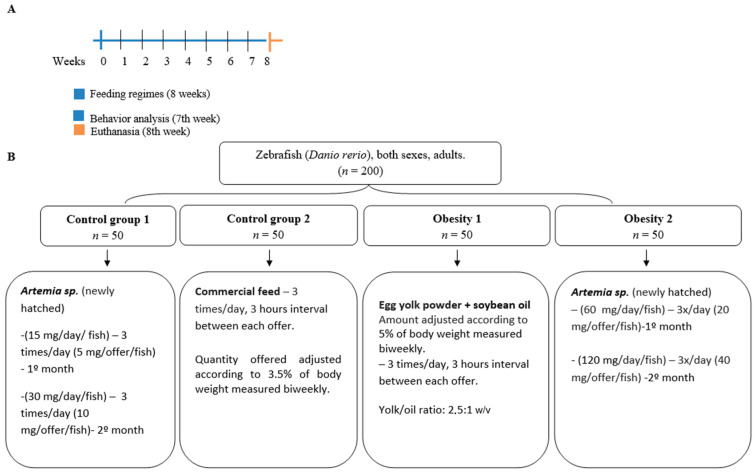
Design of the eight-week diet-induced obesity experiment. (**A**) time scale in weeks. (**B**) division of zebrafish into groups. Control group 1: fed with *Artemia* sp.; control group 2: fed with commercial fish food; obesity group 1: overfed with egg yolk added with soybean oil; and obesity group 2: overfed with *Artemia* sp.

The experimental protocol followed Oka et al. and Landgraf et al. [14,16]. Thus, a comparative study investigated the effects of different DIO models in zebrafish (*Danio rerio*). Simple randomization was used through random selection to separate the animals into groups before treatment. The animals were divided into four groups of fifty zebrafish per 8 L tank, totaling 200 adult animals. The groups were fed three times a day with 3 h intervals between each food offering and were divided as follows (Figure 1):

(1) Control group 1: fed with *Artemia* sp. (a live prey)—15 mg/day/fish (in the first month) and 30 mg/day/fish (in the second month).
(2) Control group 2: fed with commercial fish food—3.5% of the group’s average body weight.
(3) Obesity group 1: overfed with powdered egg yolk mixed with soybean oil in a proportion of 2.5 yolk to 1 oil (g/g)—5% of the group’s average body weight, simulating a high-fat diet.
(4) Obesity group 2: overfed with *Artemia* sp.—60 mg/day/fish (in the first month) and 120 mg/day/fish (in the second month).

**Figure 2:**
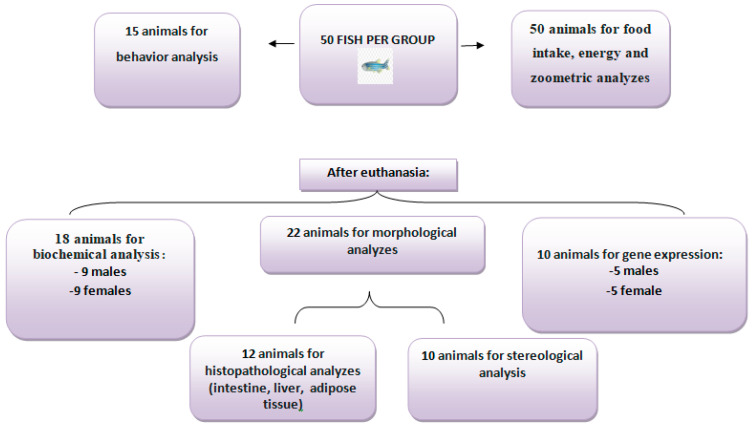
Distribution of the number of animals according to the type of analysis. All 50 animals per group were analyzed for food intake, energy intake, and zoometric analyses, and 15 were used for behavioral analysis. After euthanasia, 18 animals were used for biochemical analyses (nine males and nine females), 22 for morphological studies, and 10 for gene expression (five males and five females).

It is essential to highlight that the number of 50 animals per group was necessary due to the numerous analyses performed in the present study. Therefore, at least ten animals from each group evaluated were used in each analysis (Figure 2).

All groups were exposed to the above-described feeding regime for eight weeks. Water quality was ensured by a mechanical, biological, and chemical filtration system, and pH (~6.7), temperature (28 °C), and oxygen levels (~6 mg/L) were consistently checked and controlled, with constant renewal provided by Tecniplast’s Active Blue stand-alone water maintenance system maintaining all the ideal quality conditions for zebrafish.

### 2.3. Characterization of the Diets and Production Composition

Two diets were used to induce obesity, one composed of *Artemia* sp. and the other of powdered egg yolk and soybean oil, containing different ingredients and production compositions (Table 1 and Table 2). *Artemia* sp. was also used as food in the control diet, in a quantity considered nutritionally adequate for growth and development, as was the commercial feed Nutriflakes from Nutricon.

### 2.4. Evaluation of the Effects Associated with Obesity Induction

During the obesity induction period, the zebrafish were monitored before the start of the experiment, biweekly for weight (mg), length (cm), and body mass index (BMI-g/cm^2^) and daily for dietary intake, caloric intake, and caloric efficiency.

Before euthanasia, anxiety-like behavior was assessed, and after euthanasia, biochemical and inflammatory parameters were investigated. In addition, the livers and intestines were evaluated by histopathological analysis and visceral fat was assessed using histopathological, stereological, and inflammation-related gene expression analyses.

### 2.5. Evaluation of Dietary Consumption, Caloric Intake, and Caloric Efficiency

During the experiment (8 weeks), the animals received previously weighed portions of their respective diets. For this calculation, based on the study by Queiroz et al. [3], the initial consumption (day 1) and final consumption (day 60) of the 50 animals per group were considered after adaptation and establishment of the individual consumption pattern. With this, the variation (Δ) of dietary consumption (Equation (1)), caloric intake (Equation (2)), and caloric efficiency (Equation (3)) were calculated, with 4.184 being equivalent to the caloric conversion in kilojoules (KJ) [3].

Δ Dietary consumption = final consumed weight − initial consumed weight(1)

Caloric intake (KJ) = final dietary consumption (Kcal) × 4.184(2)

Caloric efficiency = calorie intake (KJ)/body weight variation (g)(3)

The crustacean count was estimated before and after offering food to verify the amount of *Artemia* sp. An amount of 1 mL of water from the tank after consumption was collected three times from the 8 L zebrafish-free water tank containing *Artemia* sp. After counting and feeding time, the *Artemia* sp. was returned to the 8 L tank containing the animals. Then, after 20 min, three 1 mL aliquots were collected to check the amount of remaining *Artemia* sp.

### 2.6. Body Weight, Length, and Determination of Body Mass Index (BMI)

Before the beginning (week 0) and every two weeks until the end (week 8) of the experiment, the body weight of the zebrafish was measured using a portable Brifit precision digital scale and a 50 mL beaker. The length was measured from the mouth to the end of the body using graph paper, a Petri dish with water, and the ImageMeter application (Dirk Farin Kronenstr. 49b 70174, Stuttgart, Germany). Values were recorded, and body mass indices were calculated according to Oka et al. [14] (Equation (4)). Such measurements were carried out without anesthesia.

BMI = body weight (g)/body length^2^ (cm^2^) (4)

### 2.7. Collection of Biological Material

At the end of the experimental period, the zebrafish (50 adult animals from each group) were euthanized by submersion in ice-cold water (0–4 °C) for 10 min until complete immobilization (Euthanasia guide for teaching and research animals—UNIFESP, 2019) [18], followed by immersion in clove oil (10 mL/L) to guarantee euthanasia and, consequently, the collection of biological materials for all proposed analyses. For histological analysis, animals were maintained in 10% formaldehyde. The fish intended for gene expression analysis were kept in a RNAlater from Thermo Fisher Scientific (Waltham, MA, USA) and frozen (−80 °C) until the evaluation, where they remained cooled during all procedures.

### 2.8. Analysis of Biochemical and Inflammatory Parameters

After 60 days of the experiment, the animals from the different groups were fasted (12 h). Subsequently, they were euthanized (submersion in ice water at 0–4 °C and immersion in clove oil—10 mL/L) for plasma collection. The zebrafish were macerated in a Fisherbrand sonicator (Fisher Scientific—Waltham, MA, USA), and the plasma was centrifuged and separated from the macerated parts, according to the methodology of Barcellos [19].

Then, the material was centrifuged at 12,000× *g* for 15 min at 22 °C (Centrifuge 5804r from Eppendorf—São Paulo, Brasil), collected in tubes, and used for the evaluation of TNF-α, IL-6, triglycerides (TG), glucose, insulin, and total cholesterol (CT). Biochemical analyses were performed using kits from Quibasa-Bioclin (Belo Horizonte, Brazil) in an automated manner using the colorimetric method. Samples were analyzed using commercially available immunoassay kits to measure inflammatory parameters, as proposed by Vendrame et al. [20]. A blind evaluator performed all analyses, unaware of the type of treatment received by each group of animals.

### 2.9. Microscopic Analysis of Visceral Adipose Tissue

The morphological study was carried out according to Martins et al. [21]. All the animals were collected, showing the entire abdominal and thoracic cavity for subsequent histological analysis. The organs (adipose tissue, livers, and intestines covered by visceral fat) were fixed in 10% formaldehyde. The tissues were packaged individually and cleaved, and the fixed material was dehydrated in an increasing alcohol series (70%, 80%, and 90%), cleared in xylene, impregnated, and embedded in paraffin in serial cross-sections of 3–4 μm thickness made on a manual rotary microtome (Leica RM2235, Buffalo Grove, IL, USA) cut and inserted into microscope slides stained with routine staining (hematoxylin and eosin) and covered with a coverslip.

The slides were read, emphasizing the histological organization of visceral adipose tissue. Furthermore, the presence of steatosis in the liver tissues of the groups of zebrafish was investigated using an Optical Microscope (B-1000) from Ponteranica, Italy (Optika brand). The images were captured with a 10× objective, using a digital camera for image capture (CP-20CC) and Proview Optika software 4.8.

### 2.10. Stereological Analysis of Visceral Adipose Tissue

Thirty random fields were photographed per animal (n = 10) to estimate the mean cross-sectional area of adipocytes: the volume density (Vv) of adipocytes divided by double the numerical density of adipocytes per area (2.QA) [22,23]. One 32-point test system, the STEPanizer software (compilation 1-8-0-74), was used to estimate the Vv of adipocytes (points reaching adipocytes divided by the total number of points within the test system). Conversely, the QA estimation was carried out considering the number of adipocytes within a test area (except adipocytes touching the forbidden lines) divided by the area of the test system, calculated after calibration of the STEPanizer [24].

### 2.11. Analysis of Relative Gene Expression of Inflammatory Markers

The mRNA (messenger ribonucleic acid) from tissues (visceral adipose tissue previously stored at −80 °C) of zebrafish was isolated using the PureLink™ RNA Mini Kit RNA extraction kit (Thermo Fisher Scientific, Waltham, MA, USA), following the manufacturer’s instructions.

Total RNA was quantified on the NanoDrop ND2000 spectrophotometer (Thermo Fisher Scientific, Wilmington, DE, USA) and stored at −80 °C. cDNA synthesis was performed using the PowerUp™ SYBR™ Green Master Mix kit (Thermo Fisher Science, Waltham, MA, USA), according to the manufacturer’s instructions, using a Rotor-gene Q system thermocycler (Qiagen, Redwood City, CA, USA). The cDNA was obtained and stored at −20 °C until used for expression assays.

The relative expression levels of tnf-α, il-1β, il-6, and il-10 were determined by qRT-PCR using the PowerUp™ SYBR™ Green Master Mix kit. The standard gene normalizer used to determine relative expression was beta-actin. Four primer sequences were used for gene expression (Table 3). Thermocycling conditions were a maintenance stage of 10 min at 95 °C, followed by 40 cycles of 95 °C for 15 s and 60 °C for 60 s, and finally, a melting stage of 95 °C for 15 min. The experiment was conducted in technical triplicate and biological duplicate, adhering to the Minimum Information for Publication of Quantitative Real-Time PCR Experiments (MIQE) guidelines [25].

### 2.12. Behavior Analysis—New Tank

**Figure 3:**
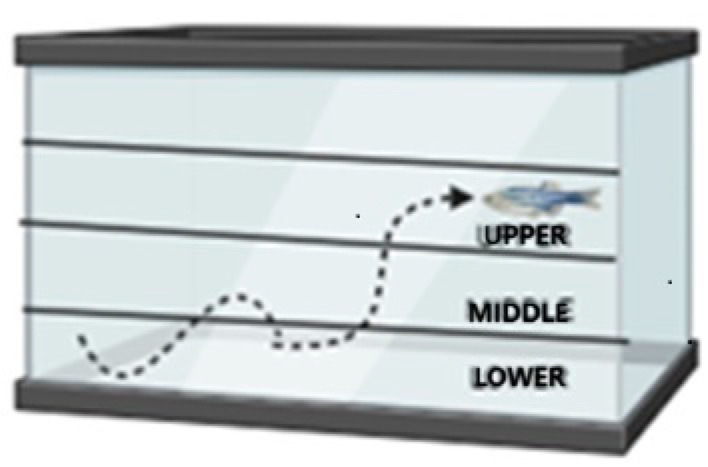
Schematic representation demonstrating the tank divisions. New tank experiment. The tank was divided into three horizontal areas of the same proportion (lower, middle, and upper).

Anxiety-like behavior was investigated through a new tank experiment following the protocol of Moreira et al. [29]. The animals (n = 15 per group) were transferred, one at a time, to a rectangular aquarium (20 cm long × 16 cm high × 12 cm wide) with up to 2.8 L and with the sides covered with white adhesive, except for the front wall, which remained transparent to allow recording. The behavior of the zebrafish was filmed for 6 min. The following parameters were considered: total distance covered, average moving speed, total time immobile, time and distance covered in the upper area, and latency to enter the area. The tank was divided into three horizontal areas of the same proportion (lower, middle, and upper) (Figure 3). The videos were analyzed using ANY-maze software 4.99z.

### 2.13. Statistical Analysis

Statistical analyses were performed using GraphPad Prism version 8.0.1 (GraphPad Software, San Diego, CA, USA). First, the data were assessed for normality using the Shapiro–Wilk test. Therefore, for the comparisons made for the biochemical and inflammatory parameters between the different treatments for the same sex, as well as for the variation in weight, length, and BMI, the data that did not present a normal distribution (non-parametric) were assessed by the Kruskal–Wallis test followed by Dunns post hoc. The remaining data that presented a normal distribution (parametric) were evaluated by ANOVA followed by Tukey post hoc.

To compare the biochemical and inflammatory data related to the evaluation between the sexes for the same treatment and the initial and final zoometric data for the same treatment, Student’s *t*-test was used for data with a parametric distribution, and the Wilcoxon test for data with a non-parametric distribution. A significance level of 0.05 was adopted for all statistical analyses.

ANOVA was used to compare the stereological analysis data regarding adipocyte volume density, followed by Tukey’s post hoc test. For the mean cross-sectional area, the Kruskal–Wallis test was performed, followed by Dunns’ post hoc test. Regarding investigating anxiety-like behavior, data were evaluated using the Kruskal–Wallis test and Dunns’ post hoc test.

## 3. Results

### 3.1. Dietary Consumption, Caloric Intake, and Caloric Efficiency

The values of dietary consumption, intake, and caloric efficiency presented are considered the representative variation per group (*n* = 50 animals) (Table 4). It was observed that the dietary intake of obesity group 2, composed of zebrafish overfed with *Artemia* sp., showed the greatest variation in consumption (0.060 g). However, despite being considered a control group, the zebrafish in control group 1 consumed almost four times more than those in control 2.

Regarding caloric intake and efficiency, it was possible to observe that the animals that presented the greatest variation in dietary intake also presented the greatest variation in caloric intake. Furthermore, the group of zebrafish with DIO that showed the greatest variation in caloric efficiency was obesity group 2, in addition to being the one that presented the highest intake and greatest variation in weight. Zebrafish in obesity group 2 had twice the caloric efficiency of those in obesity group 1 (Table 4).

For the values presented, the initial consumption (day 1) of the 50 animals per group and the final consumption (day 60) of the same were considered. Thus, the variation (Δ) of dietary consumption and, subsequently, intake and caloric efficiency were obtained.

Control group 1: fed with *Artemia* sp. (first month: 15 mg/day/fish and in the second month 30 mg/day/fish); control group 2: fed with commercial food (3.5% of the group’s average weight); obesity group 1: overfed with powdered egg yolk mixed with soybean oil (2.5:1 *w*/*w*)—5% of average body weight; obesity group 2: overfed with *Artemia* sp. in the first month (60 mg/day/fish) and in the second month (120 mg/day/fish).

### 3.2. Body Weight, Length, and Body Mass Index (BMI)

**Figure 4:**
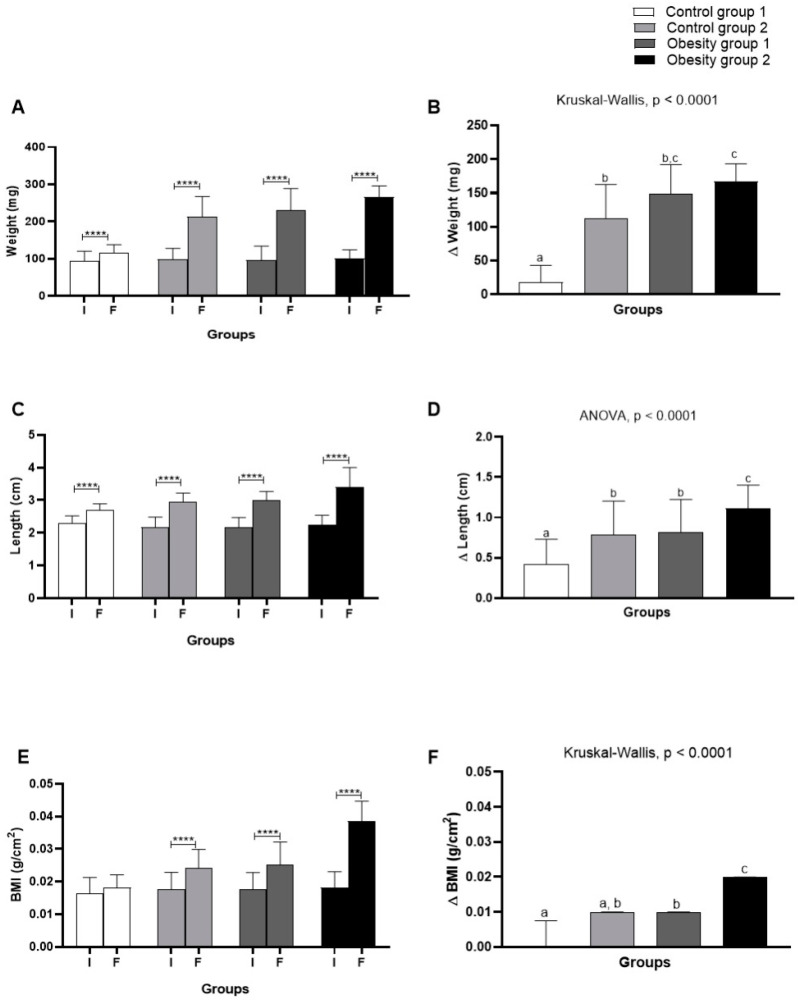
Comparison of initial (I) and final (F) assessment and variation (Δ) of body weight, length, and body mass index (BMI) of adult zebrafish (three months) with diet-induced obesity (DIO) for eight weeks. (**A**) Weight; (**B**) weight variation; (**C**) length; (**D**) length variation; (**E**) BMI; (**F**) BMI variation. Values are expressed as mean (standard deviation). *n* = 50 adult animals per group. Letters a, b, and c represent statistical differences in the comparison between groups. Equal letters indicate no significant difference between the groups evaluated for each parameter (Asterisks: **** *p* = 0.0001). Control group 1: fed with *Artemia* sp. (first month: 15 mg/day/fish and in the second month 30 mg/day/fish); control group 2: fed with commercial food (3.5% of the group’s average weight); obesity group 1: overfed with powdered egg yolk mixed with soybean oil (2.5:1 *w*/*w*)—5% of average body weight; obesity group 2—overfed with *Artemia* sp. in the first month (60 mg/day/fish) and in the second month (120 mg/day/fish). BMI: body mass index.

Comparing body weight, length, and BMI (Figure 4) evaluated at the beginning and end of the experiment, as well as considering the variation (Δ) of these parameters, an adequate gain in body weight was found for zebrafish according to their growth demands. Considering control groups 1 and 2, fed with *Artemia* sp. and commercial feed, respectively, the variation in weight gain was greater (Figure 4B) in control group 2 in the range of 72.25–162.8 mg (*p* < 0.0001). The groups of zebrafish with DIO had an increasing and significant weight gain in the range of 143.5–193.5 mg (*p* < 0.0001), regardless of diet.

**Figure 5:**
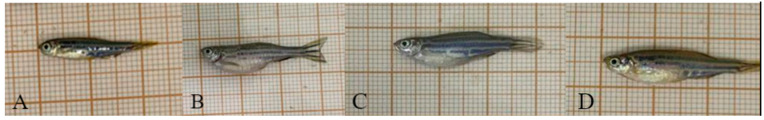
Visual comparison of zebrafish with DIO. (**A**) Control group 1: fed with *Artemia* sp. (first month: 15 mg/day/fish and in the second month 30 mg/day/fish); (**B**) Obesity group 1: overfed with powdered egg yolk mixed with soybean oil (2.5:1 *w*/*w*); (**C**) Control group 2: fed with commercial food (3.5% of the group’s average weight); (**D**) Obesity group 2—overfed with *Artemia* sp. in the first month (60 mg/day/fish) and in the second month (120 mg/day/fish).

It is worth noting that obesity group 2 presented the highest weight and length and greater variation. Likewise, the BMI results indicated obesity for fish belonging to obesity groups 1 = 0.0253 g/cm^2^ (0.021–0.020; *p* < 0.0001) and 2 = 0.024 g/cm^2^ (0.022–0.026; *p* < 0.0001) (Figure 4E). Therefore, the results showed that DIO effectively induced obesity in zebrafish. On the other hand, the diet consisting of commercial food, even in quantities considered adequate for the growth and development of zebrafish, also led the animals to obesity (Figure 5).

### 3.3. Biochemical and Inflammatory Parameters

**Figure 6:**
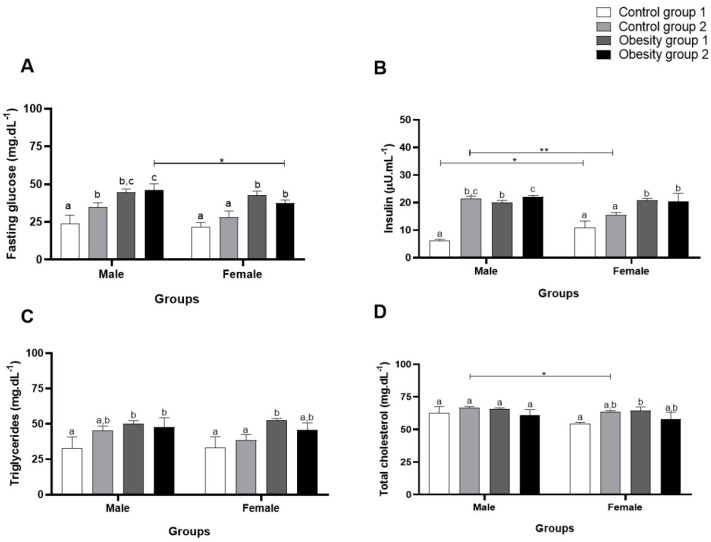
Concentrations of biochemical parameters of adult male and female zebrafish (three months) with diet-induced obesity (DIO) for eight weeks. Values are expressed as mean (standard deviation). *n* = 50 adult animals per group. Letters a, b and c represent statistical differences in the comparison between groups. Equal letters indicate no significant difference between the groups evaluated for each parameter. Asterisks: * *p* < 0.05; ** *p* < 0.001. Control group 1: fed with *Artemia* sp. (first month: 15 mg/day/fish and in the second month 30 mg/day/fish); control group 2: fed with commercial food (3.5% of the group’s average weight); obesity group 1: overfed with powdered egg yolk mixed with soybean oil (2.5:1 *w*/*w*)—5% of average body weight; obesity group 2—overfed with *Artemia* sp. in the first month (60 mg/day/fish) and in the second month (120 mg/day/fish). (**A**): results of Fasting glucose; (**B**): results of insulin; (**C**): results of triglycerides; (**D**): results of total cholesterol.

Regarding biochemical parameters, fasting plasma glucose, for both sexes, presented significantly higher values in the groups overfed to cause DIO compared to the control groups (obesity 1: 44.56 (2.22) mg/dL/*p* = 0.0009; obesity 2: 46.11 (4.14) mg/dL/*p* = 0.0005) (Figure 6A). For plasma insulin, groups with diet-induced obesity had significantly higher concentrations (obesity 1: 20.06 (0.68) µU/mL/*p* < 0.0001, and obesity 2: 21.99 (0.61) µU/mL/*p* < 0.0001) than those of control 1 (Figure 6B).

Triglyceride concentrations also showed a significant increase for both sexes in obesity groups 1 and 2 (obesity 1: 50.33 (2.08) mg/dL/*p* = 0.02 and obesity 2: 47.67 (6.73) mg/dL/*p* = 0.04) compared to control groups 1 and 2 (Figure 6C). However, for total cholesterol (Figure 6D), there was no significant difference between males, while females in obesity group 1 showed a significantly higher concentration (64.44 (2.77) md/dL/*p* = 0.04) compared to control group 1.

**Figure 7:**
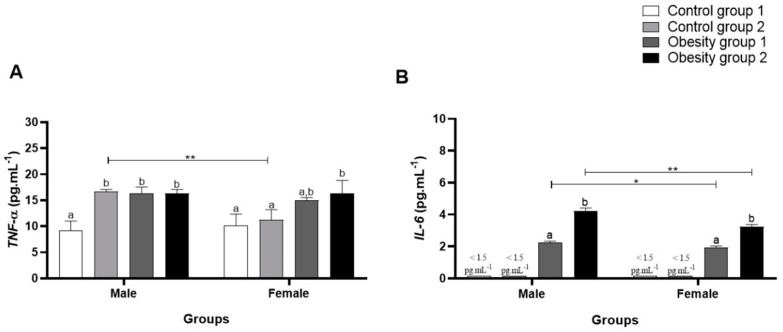
Plasma concentrations of inflammatory parameters of adults male and female zebrafish (three months) with diet-induced obesity (DIO) for eight weeks. Values are expressed as mean (standard deviation), n = 9 adult animals per group and sex. Letters a and b represent statistical differences in the comparison between groups. Equal letters indicate no significant difference between the groups evaluated for each parameter (Asterisks: * *p* < 0.05; ** *p* < 0.01). Control group 1: fed with *Artemia* sp. (first month: 15 mg/day/fish and in the second month 30 mg/day/fish); control group 2: fed with commercial food (3.5% of the group’s average weight); obesity group 1: overfed with powdered egg yolk mixed with soybean oil (2.5:1 *w*/*w*)—5% of average body weight; obesity group 2—overfed with *Artemia* sp. in the first month (60 mg/day/fish) and in the second month (120 mg/day/fish). (**A**): results of TNF-α: tumor necrosis factor alpha; (**B**): results of IL-6: interleukin-6.

When evaluating plasma inflammatory parameters, it was observed that TNF-α was significantly higher in females and males from the obesity 1 and obesity 2 groups (obesity 1: 16.39 (1.17) pg/mL/*p* = 0.0003, and obesity 2: 16.33 (0.79) pg/mL/*p* = 0.0004) than control group 1 (Figure 7A).

Furthermore, the data obtained for IL-6 (Figure 7B) were significantly higher in zebrafish from obesity groups 1 and 2, being even higher in females and males from obesity group 2 (overfed with *Artemia* sp.) (4.24 (0.18) pg/mL/*p* < 0.0001). However, plasma IL-6 concentrations in males in obesity groups 1 and 2 compared to controls 1 and 2 were even higher. For conventionally fed zebrafish (control groups 1 and 2), plasma IL-6 concentrations were below the kit’s minimum detection values, making statistical analysis impossible. The results indicate that the obesity induction diets tested generated inflammation in zebrafish and, in this study, for IL-6, sex influenced the outcome.

### 3.4. Relative Gene Expression of Inflammatory Markers of Visceral Adipose Tissue in Zebrafish with Obesity

**Figure 8:**
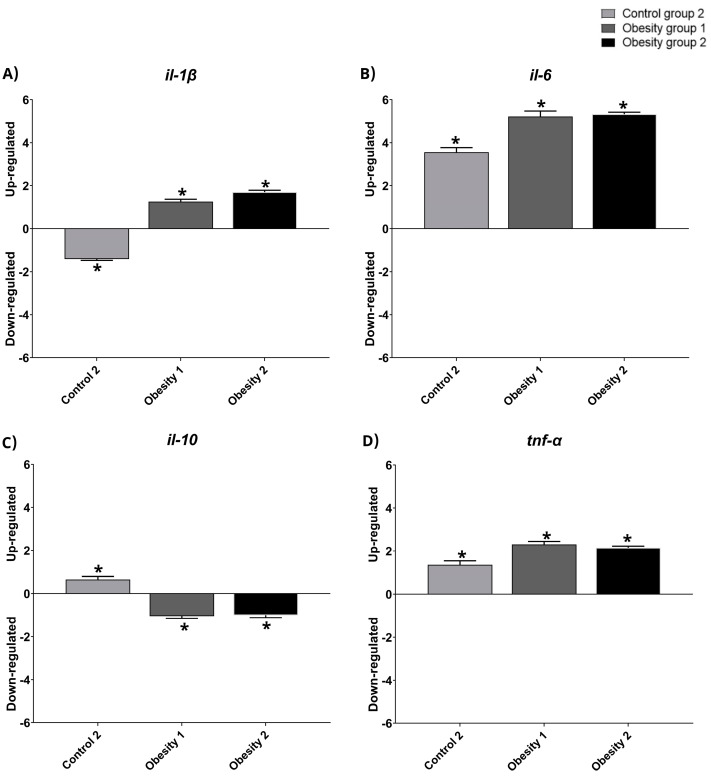
Relative gene expression values of inflammatory markers in visceral adipose tissue in adult male zebrafish (three months) with diet-induced obesity (DIO) for eight weeks. Qualitative data and normalization with control group 1. Control group 2: fed with commercial food (3.5% of the group’s average weight); obesity group 1: overfed with powdered egg yolk mixed with soybean oil (2.5:1 *w*/*w*)—5% of average body weight; obesity group 2—overfed with *Artemia* sp. in the first month (60 mg/day/fish) and in the second month (120 mg/day/fish). Asterisks indicate significant statistical differences. (**A**): *il-1 β*: interleukin 1 beta; (**B**): *il-6*: interleukin 6; (**C**): *il-10*: interleukin 10; (**D**): *tnf-α:* tumor necrosis factor alpha. Down-regulated: low gene expression. Up-regulated: high gene expression.

**Figure 9:**
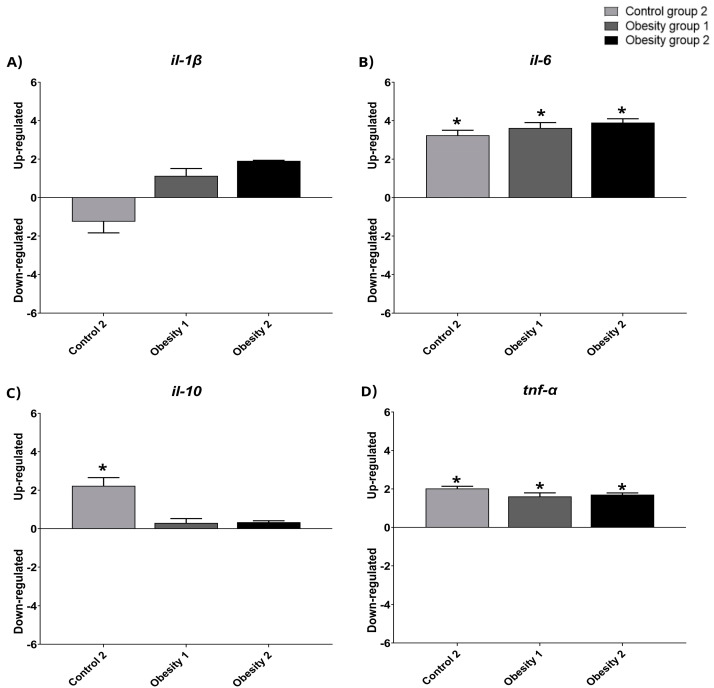
Relative gene expression values of inflammatory markers in visceral adipose tissue in adult female zebrafish (three months) with diet-induced obesity (DIO) for eight weeks. Qualitative data and normalization with control group 1. Control group 2: fed with commercial food (3.5% of the group’s average weight); obesity group 1: overfed with powdered egg yolk mixed with soybean oil (2.5:1 *w*/*w*)—5% of average body weight; obesity group 2—overfed with *Artemia* sp. in the first month (60 mg/day/fish) and in the second month (120 mg/day/fish). Asterisks indicate significant statistical differences. (**A**): *il-1 β*: interleukin 1 beta; (**B**): *il-6*: interleukin 6; (**C**): *il-10*: interleukin 10; (**D**): *tnf-α*: tumor necrosis factor alpha. Down-regulated: low gene expression. Up-regulated: high gene expression.

The relative gene expression data were qualitative and normalized with control group 1 (Figure 8 and Figure 9). The results indicated the presence of inflammation in males from obesity induction groups 1 and 2, characterized by higher relative expression of il-1-β, il-6, and tnf-α. Furthermore, females in obesity groups 1 and 2, compared to control group 2, also showed more inflammation.

### 3.5. Histopathological Aspects of Visceral Adipose Tissue in Zebrafish with Obesity

**Figure 10:**
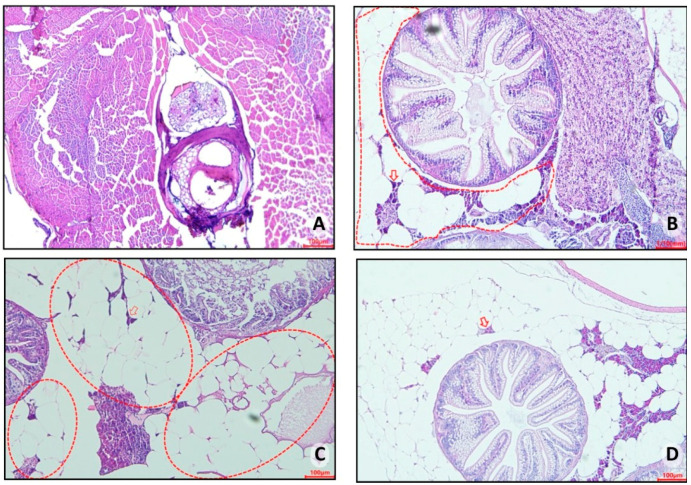
Representative photomicrograph, at 10× using a 100 µm objective, of visceral adipose tissue from adult zebrafish (three months) with diet-induced obesity (DIO) for eight weeks. In control group 2, an accumulation of unilocular adipocytes was observed involving organs located in the abdominal cavity (dashed circle) with the presence of macrophages (red arrow) (**B**). The fish in obesity group 1 showed an extensive area of unilocular adipose tissue distributed in the abdominal cavity (red outline), in addition to a crown of macrophages surrounding the adipocytes (red arrow) (**C**). Zebrafish from obesity group 2 presented the highest number of unilocular adipocytes surrounded by macrophages (red arrow). (**A**) Control group 1: fed with *Artemia* sp. (first month: 15 mg/day/fish and in the second month 30 mg/day/fish); (**B**) control group 2: fed with commercial food (3.5% of the group’s average weight); (**C**) obesity group 1: overfed with powdered egg yolk mixed with soybean oil (2.5:1 *w*/*w*)—5% of average body weight; (**D**) obesity group 2—overfed with *Artemia* sp. in the first month (60 mg/day/fish) and in the second month (120 mg/day/fish).

Histopathological analyses of control group 1 (fed *Artemia* sp.) showed no important presence of fatty tissue in the animals, as seen in this group’s representative photomicrograph (Figure 10A). However, in control group 2 (fed with commercial food), an accumulation of unilocular adipocytes was observed involving organs located in the abdominal cavity (dashed circle) with the presence of macrophages (red arrow) (Figure 10B). The fish in obesity group 1 (overfed with powdered egg yolk mixed with soybean oil) showed an extensive area of unilocular adipose tissue distributed in the abdominal cavity (red outline), in addition to a crown of macrophages surrounding the adipocytes (red arrow) (Figure 10C). Zebrafish from obesity group 2 (overfed with *Artemia* sp.) were those with the highest number of unilocular adipocytes surrounded by macrophages (red arrow) (Figure 10D).

### 3.6. Visceral Body Fat: Stereological Analysis

**Figure 11:**
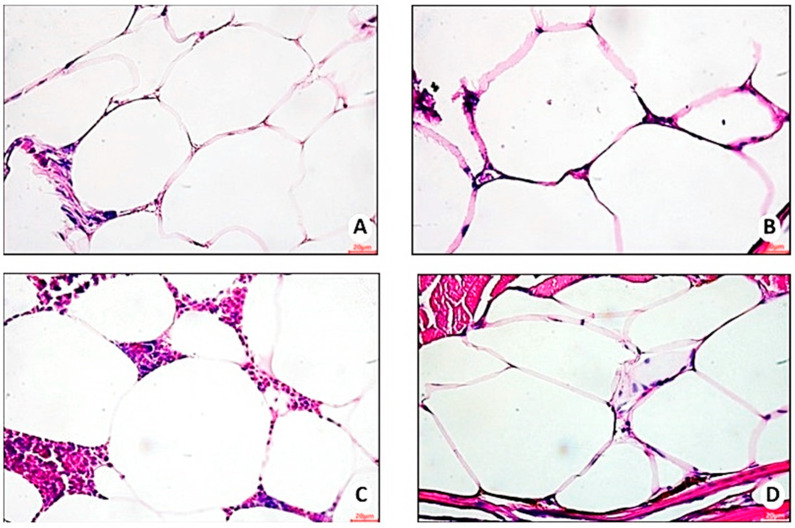
Representative photomicrograph, at 40× using a 20 µm objective, of hypertrophied adipocytes in visceral adipose tissue of zebrafish with diet-induced obesity (DIO) for eight weeks (*n =* 10 per group). (**A**) Control group 1: fed with *Artemia* sp. (first month: 15 mg/day/fish and in the second month 30 mg/day/fish); (**B**) control group 2: fed with commercial food (3.5% of the group’s average weight); (**C**) obesity group 1: overfed with powdered egg yolk mixed with soybean oil (2.5:1 *w*/*w*)—5% of average body weight; (**D**) obesity group 2—overfed with *Artemia* sp. in the first month (60 mg/day/fish) and in the second month (120 mg/day/fish).

**Figure 12:**
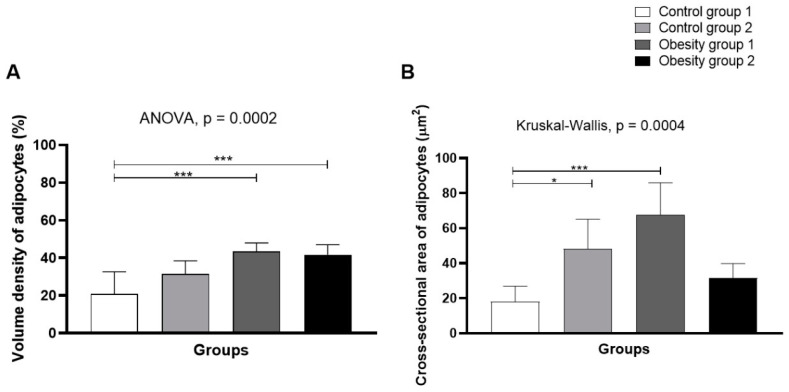
Volume density (**A**) and mean cross-sectional area of adipocytes (**B**) from visceral adipose tissue of zebrafish adults (three months) with diet-induced obesity (DIO) for eight weeks. Control group 1: fed with *Artemia* sp. (first month: 15 mg/day/fish and in the second month 30 mg/day/fish); control group 2: fed with commercial food (3.5% of the group’s average weight); obesity group 1: overfed with powdered egg yolk mixed with soybean oil (2.5:1 *w*/*w*)—5% of average body weight; obesity group 2—overfed with *Artemia* sp. in the first month (60 mg/day/fish) and in the second month (120 mg/day/fish). Asterisks: * *p* < 0.05; *** *p* < 0.0001.

Stereology (Figure 11) indicated higher volume density (43.6% (4.40)/*p* = 0.0002) and mean sectional area of adipocytes in obesity group 1 (fed *Artemia* sp.) (67.8 (18, 1) µm2/*p* = 0.0004—Figure 12A,B), followed by control group 2.

### 3.7. Histopathological Analysis in Hepatocytes and Intestines of Zebrafish with Obesity

**Figure 13:**
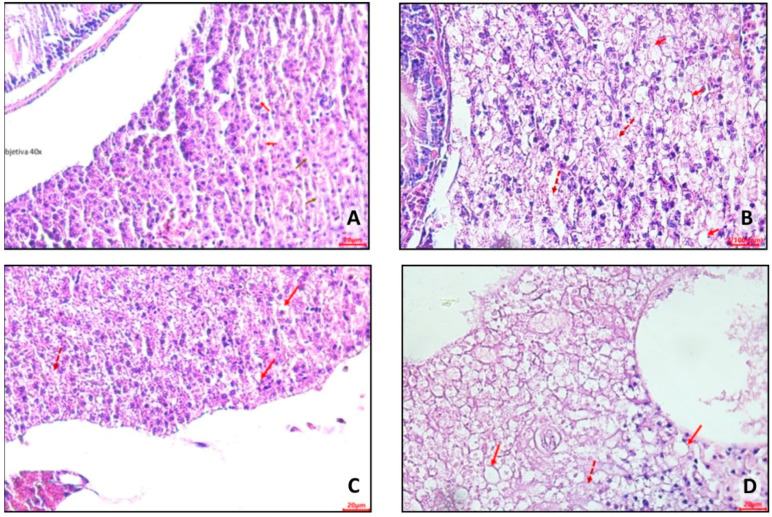
Representative photomicrographs, at 40× using a 20 µm (**A**,**C**,**D**) and 100 µm (**B**) objective, of hepatocytes from adult zebrafish (three months) with diet-induced obesity (DIO) for eight weeks. (**A**) Control group 1: fed with *Artemia* sp. (first month: 15 mg/day/fish and in the second month 30 mg/day/fish); (**B**) control group 2: fed with commercial food (3.5% of the group’s average weight); (**C**) obesity group 1: overfed with powdered egg yolk mixed with soybean oil (2.5:1 *w*/*w*)—5% of average body weight; (**D**) obesity group 2—overfed with *Artemia* sp. in the first month (60 mg/day/fish) and in the second month (120 mg/day/fish). The red arrows indicate hepatocytes with a large amount of lipid deposits in the cytoplasmic location, and the dashed hepatocytes have areas of necrosis and the absence of nuclei.

In control group 1 (Figure 13A), hepatocytes were evident with a small amount of lipid deposits in a cytoplasmic location (clear halos—red arrow) and areas of necrosis with the absence of nuclei (brown arrow). In control group 2 (Figure 13B), it was possible to observe hepatocytes containing lipid deposits in a cytoplasmic location (microvesicular steatosis). Furthermore, areas of necrosis with the absence of nuclei were identified (dashed arrow).

In obesity group 1 (Figure 13C), hepatocytes with a large amount of lipid deposits in cytoplasmic location (clear halos—red arrow) and areas of necrosis with absence of nuclei (dashed arrow) were observed. In obesity group 2 (Figure 13D), hepatocytes were visualized with a large amount of lipid deposits (microvesicular and macrovesicular steatosis) in a cytoplasmic location (clear halos—red arrow), along with areas of necrosis (absence of nuclei—dashed arrow).

**Figure 14:**
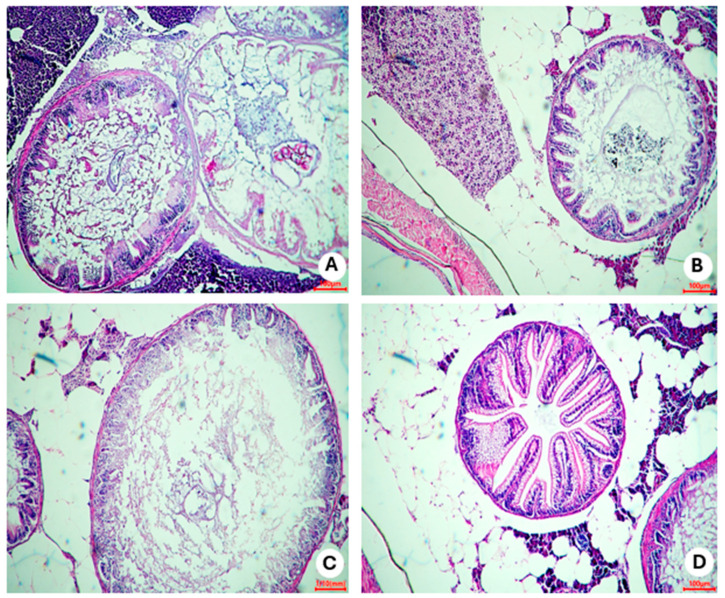
Representative photomicrographs, at 10× using a 100 µm objective, of areas of the intestine of zebrafish adults (three months) with diet-induced obesity (DIO) for eight weeks. (**A**) Control group 1: fed with *Artemia* sp. (first month: 15 mg/day/fish and in the second month 30 mg/day/fish); (**B**) control group 2: fed with commercial food (3.5% of the group’s average weight); (**C**) obesity group 1: overfed with powdered egg yolk mixed with soybean oil (2.5:1 *w*/*w*)—5% of average body weight; (**D**) obesity group 2—overfed with *Artemia* sp. in the first month (60 mg/day/fish) and in the second month (120 mg/day/fish).

In representative photomicrographs of slides of intestine portions (Figure 14), inflammatory infiltrate was verified in obesity group 2 (Figure 14C). Disorganized areas in the structure of the intestinal villi were observed. On the other hand, preserved cells in other portions of the intestine were visualized, with no apparent impairment of function in this organ.

### 3.8. Behavior Analysis—New Tank

**Figure 15:**
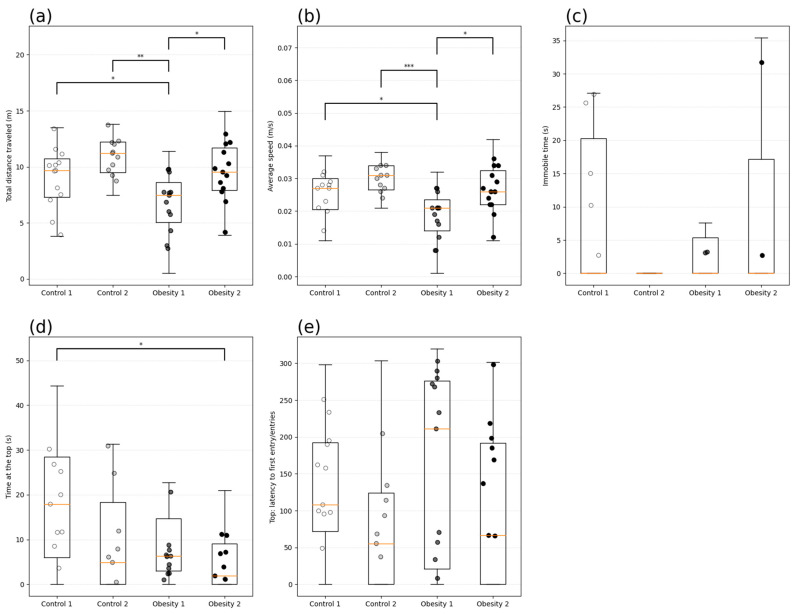
Behavioral analysis of anxiety by the new tank test of adult male and female zebrafish (three months) with diet-induced obesity (DIO) for eight weeks. Data are expressed as medians (interquartile distance) analyzed using the Kruskal–Wallis test. Each circle represents an individual animal (n = 15). Control group 1: fed with *Artemia* sp. (first month: 15 mg/day/fish and in the second month 30 mg/day/fish); control group 2: fed with commercial food (3.5% of the group’s average weight); obesity group 1: overfed with powdered egg yolk mixed with soybean oil (2.5:1 *w*/*w*)—5% of average body weight; obesity group 2—overfed with *Artemia* sp. in the first month (60 mg/day/fish) and in the second month (120 mg/day/fish). Asterisks indicate significant statistical differences: * *p* < 0.05; ** *p* < 0.01; *** *p* < 0.0001. (**a**) Total distance traveled (m); (**b**): Average speed (m/s); (**c**) Immobile time (s); (**d**): Time at the top (s); (**e**) Top: latency to first entry.

In analyzing behavior associated with the anxious profile, the time (s) of immobility and exploration (m) of the top of the tank was evaluated. Zebrafish from obesity group 1 showed anxiety-like behavior, identified by the longer latency to enter the upper area (Figure 15e) (184.19 (110 s), shorter total distance covered (Figure 15a) (*p* = 0.009), and lower average speed (Figure 15b) (*p* = 0.0007) than the other groups investigated.

All the presented results indicate that the different diets could cause complications from excessive weight gain.

## 4. Discussion

In this study, it was possible to compare the administration of different DIO models, considering the differences between the sexes of zebrafish in inflammatory and biochemical parameters, in addition to the effects related to zoometric, physiological, histological, molecular, and behavioral parameters (such as anxiety). Thus, a better understanding of the development of obesity and its complications was allowed. First, it is important to highlight that caloric efficiency is related to caloric intake and weight variation. Thus, the data obtained for caloric efficiency showed weight gain in all groups investigated. However, the evaluation of dietary intake showed that zebrafish in obesity group 2 presented greater variation in consumption. It is known that the *Artemia* sp. consumed by this group is a food with high dietary acceptance and is recommended in the guidelines for feeding laboratory-raised zebrafish [8,15,17]. On the other hand, zebrafish in control group 2 fed commercial food for ornamental fish showed less variation in consumption, possibly because the food offered contained a significant amount of carbohydrate in its composition, which is not specified for feeding zebrafish, despite being recommended for use in vivarium [8].

The high palatability and acceptance of *Artemia* sp. observed in this study, recorded by the absence of leftovers during fish feeding, allowed a greater increase in the amount offered during the experiment. Considering studies published in the literature [9,14,15,16,17] involving DIO, also using *Artemia* sp., it is believed that the greater caloric efficiency and dietary consumption presented by the control 1 and obesity 2 groups are related to the greater acceptance of this type of live food. Therefore, it is very advantageous to use this food, especially in DIO protocols.

In addition, *Artemia* sp. is considered a caloric food [9,14,15,16,17], having been the most consumed compared to the other diets offered. It was also noted that obesity group 2 presented the greatest caloric intake and variation in weight, in addition to the variation in consumption and caloric efficiency.

Meanwhile, control group 2, because it had the lowest dietary intake and because commercial feed was the least caloric food, had the lowest caloric intake. On the other hand, significant weight gain was evidenced for this group compared to control 1 (*Artemia* sp.). The carbohydrate content can explain this since this animal does not require substantial amounts of this macronutrient for its growth and development [8]. Therefore, it can be highlighted that even a diet not offered as overfeeding can cause changes due to the imbalance in its nutritional composition.

Regarding the overfeeding that was prepared using the high-fat mixture of egg yolk and soybean oil (offered to obesity group 1), in addition to causing some expected complications, it proved to be a highly palatable food. It presented 100% acceptance in all offers, being the diet with the highest caloric intake. Thus, this study demonstrated that when the diet is nutritionally adequate and offered in an adjusted quantity (control group 1—*Artemia* sp.) only for growth and development, even if the animals present excellent caloric efficiency, this will not necessarily result in weight gain above what would be expected for these animals. On the other hand, as demonstrated by control group 2 (commercial feed), a diet that is not nutritionally adequate, even when offered in a sufficient quantity, may impact the animals’ weight gain.

In addition, it was possible to note that even nutritionally balanced foods, such as *Artemia* sp., if ingested in excess, can cause weight gain and complications resulting from excess adiposity. This has been verified in other studies [9,14,16,17], and it was proven that healthy foods consumed in excess can cause complications resulting from obesity. Regarding the growth of zebrafish, it was impossible to perceive a difference in the influence of the different foods offered since all diets led the animals in the different groups to satisfactory growth according to the comparison of their variation in length. However, obesity group 2 (*Artemia* sp.) showed greater growth than the other groups investigated (*p* < 0.0001).

In this study, the biochemical parameters of glucose, insulin, cholesterol, and triglycerides were evaluated to compare the obesity-inducing diets, investigating the influence more specifically on these parameters and, consequently, on the emergence of several related diseases and whether there were differences between the sexes. Thus, it was found that the diets used to induce obesity were capable of causing changes in fasting blood glucose and insulin, which could result in the development of diabetes mellitus. It is already known from the literature that zebrafish show a significant response to the increase in insulin under conditions of hyperglycemia [22]. Regarding the comparison between the sexes, it is possible to highlight that, in obesity group 2, the blood glucose value was higher for males. There was a significant difference in blood glucose for females, which was higher in the groups with DIO than in the control groups. However, it is essential to highlight that males are the best option for induced disorders related to fasting blood glucose and plasma insulin concentration because they present higher values.

The compositions of the diets used in this study favor lipid accumulation. Egg yolk with soybean oil is a hyperlipidemic mixture, and *Artemia* sp. consumed in excess also provides a high fat content. These diets led to higher concentrations of triglycerides and cholesterol and could, therefore, be considered excellent inducers of dyslipidemia for both sexes. However, male zebrafish presented higher values for plasma cholesterol concentrations than females.

For triglycerides, it was found that females in obesity group 1 had higher concentrations than the control groups. Other studies have also found such changes in zebrafish, but few have evaluated the difference between the sexes [6,23]. In the present study, it was found that there are differences between the sexes related to complications resulting from obesity. This result corroborates the study by Oka et al. [14], who also observed differences between the sexes in the lipid profiles of zebrafish. Given these findings related to lipid profiles, it can be stated that zebrafish are an adequate experimental model to investigate lipid metabolism and clarify the molecular bases of the development of hyperlipidemia disorders in humans [30].

The association between obesity and inflammatory parameters was verified, and it was observed that obesity groups 1 and 2 presented elevations in plasma concentrations of TNF-α and IL-6. Comparing the sexes, males presented higher and more significant values for both DIO groups than females for IL-6. It is known that a high-fat diet generates low-grade chronic inflammation. In addition, increased body fat causes chronic inflammation and elevation in hormone levels [31,32,33]. However, in this study, this relationship seemed to have a close association with sex. In humans, males may present some inflammatory markers that are more significant than those of women, which may stimulate the development of some cardiovascular diseases [33].

The results obtained for the analysis of relative gene expression, even though they were only qualitative, were consistent with the results of the plasma inflammatory parameters, indicating the greater expression of inflammatory markers in the groups of animals (obesity 1 and 2) that also presented the highest plasma concentrations of the same. This increase was greater in male animals but was also evident in females. Landgraf et al. [16] also found a significant increase in the inflammatory state in males, and Banerjee et al. [34] confirmed the association of weight gain with increased inflammation without comparing the sexes. However, a limitation of the study was not having carried out an assessment of the expression of obesity-related genes such as family peroxisome proliferator-activated receptor (PPAR), adipocyte fatty acid binding protein (AFABP, also known as aP2 and FABP4), and stearoyl-CoA desaturase (SCD). Nonetheless, the study makes comparisons in several other aspects.

In obesity, adipose tissue is an important endocrine organ that acts as a calorie reservoir. In conditions of overfeeding, this organ stores excess lipids, mainly in the form of neutral lipids [35,36]. In obesity, a disordered change occurs in this adipose tissue [14,36,37]. When there is an excessive intake of calories in the diet, especially from fats and carbohydrates, the body stores excess energy in lipids in adipocytes. Lipids are stored in droplets inside adipose cells, increasing their size and volume [35]. Zebrafish adipose tissue is similar in appearance and function to the white adipose tissue found in mammals. It shares many of the same markers as mammalian white adipose tissue and reacts to changes in energy balance in a way that suggests it performs similar functions. Additionally, as in humans, zebrafish have distinct types of adipose tissue, including visceral and subcutaneous types, which have been identified with specific roles and locations [37].

This study identified extensive visceral adipose tissue areas in zebrafish from control group 2 and the groups with DIO with macrophage infiltration. However, immunohistochemistry (IHC) was not used in this analysis to further clarify the conclusion, which is a limitation of this study. These groups had the greatest weight gain, the highest number of complications, and the highest concentrations of inflammatory markers in the plasma. These facts confirm the association of obesity with inflammation [26].

Regarding the stereological aspects of fatty tissue, this study highlighted obesity group 1 (fed with egg yolk and soybean oil), proving that a high-fat diet effectively promotes an increase in the mean cross-sectional area and volume density of adipocytes. Furthermore, this group also presented altered inflammatory parameters, as observed in other studies on obesity induction [14,16].

In addition to the histopathological and stereological aspects of fatty tissue, fat accumulation, necrosis, and inflammation were observed in the hepatocytes of animals in the control 2, obesity 1, and obesity 2 groups. These findings were also observed in other DIO studies, which were accompanied by changes in the expression of genes recognized as markers for lipid metabolism, inflammation, and necrosis [12,14,16].

Regarding the histopathological analyses of the intestine, inflammatory infiltrates were identified mainly in the control 2 and obesity 1 groups. Intestinal villi were also compromised in obesity group 1 (fed with egg yolk and soybean oil). It is already known that a high-fat diet is associated with an intestinal inflammatory state [22,34]. In obesity group 2, few inflammatory infiltrates were found, indicating that even with significant weight gain, this organ remained preserved in these animals.

Studies of the changes associated with obesity are important to understand the pathophysiology and assist in evaluating treatments. Experimental animal models are promising and necessary, as they are reproducible without major difficulties. This is a pioneering comparative study of complications caused by obesity-inducing diets in zebrafish, evaluating the most diverse parameters that bring significant comparisons between males and females.

For the high-fat diet, the present study demonstrated that the group overfed with egg yolk and oil (obesity group 1) presented an extensive visceral adipose tissue area with inflammation, weight gain, and biochemical alterations. However, it was observed that the greatest alterations resulting from obesity in this group were associated with triglyceride levels. Furthermore, it was found that when *Artemia* sp. was offered in larger quantities to induce obesity (obesity group 2), it caused complications of this disease, and it was observed that, for male zebrafish, these consequences were even greater, mainly for glycemia, total cholesterol, and inflammatory parameters. Therefore, it was observed that sex influenced all parameters in which the difference between the sexes was investigated.

Further to the issue of weight gain caused by obesity-inducing diets, one of the associations resulting from obesity is the relationship between weight gain and anxious behavior. Anxiety is affected by several factors, but it is known that there is a strong relationship between excess food intake, mainly characterized by the consumption of a high-fat diet, through the down-regulation of genes associated with appetite [12,38]. In other studies, prolonged exposure to a high-fat diet led to significant changes in the regulation of inflammation, apoptosis, cell proliferation, and the activation of glial cells and neurogenesis in zebrafish [39,40,41].

It is already known that the blood–brain barrier (BBB) is affected by obesity [42]. Adipocyte hyperplasia and hypertrophy cause tissue hypoxia that induces the secretion of inflammatory macrophages and inflammatory cytokines into the circulation. These inflammatory markers can cross the BBB, activate more macrophages in the brain, and induce inflammation in the central nervous system (CNS). However, little is known about neuroinflammation, and more neuroimaging research in humans is needed to validate the results already demonstrated in animals further [42,43,44].

In this study, it was possible to observe that the high-calorie and high-fat diet composed of egg yolk with soybean oil (obesity group 1) caused anxiety-like behavior, revealed by the parameters analyzed in the new tank test. Meanwhile, the zebrafish in control groups 1 and 2 that were not overfed showed smaller changes in these parameters. However, it is noteworthy that the zebrafish in control group 2 (fed with commercial food) also gained significant weight and did not present an anxious profile, suggesting that overfeeding may be more related to anxiety. However, this study only performed one test that investigated anxiety-like behavior, and other analyses are necessary to infer the influence of a diet on anxious behavior.

Thus, it was noted that diets might have inherent, unique characteristics that influence food consumption or efficiency and biochemical, morphological, and behavioral changes. Therefore, it is essential to evaluate what is intended to be investigated to define the diet type with the greatest potential to achieve the proposed objective. Thus, this animal model is indicated for evaluating the effects of foods and therapeutic agents, as well as for evaluating the possibilities of prevention and treatment in the development of obesity.

## 5. Conclusions

The obesity-inducing diets evaluated in this study were capable of causing obesity and associated complications. However, each type of diet presented inherent characteristics and may be indicated for specific investigations. In this sense, it was found that overfeeding with *Artemia* sp. promoted the greatest feed efficiency and induced the greatest weight gain, in addition to the greatest biochemical and inflammatory changes and hepatic steatosis. However, overfeeding with the high-fat diet (egg yolk with soybean oil) also caused significant changes, emphasizing increased plasma triglyceride concentrations, intestinal inflammation, hepatic steatosis, increased cross-sectional area and volume density of adipocytes, and behavioral changes. Furthermore, sex influenced some evaluated parameters, emphasizing males who presented inflammatory changes and higher fasting blood glucose levels.

Thus, the present study may help to establish a protocol for inducing obesity and associated diseases in zebrafish, aiming at evaluating new therapies or adjuvant treatments with potential anti-obesity effects.

## Figures and Tables

**Table 1:** Ingredients present in obesity-inducing diets.

DIO	Ingredients
*Artemia* sp. ^1^	*Artemia* sp. (newly hatched nauplii)
Commercial Fish food (Nutriflakes from Nutricon) ^2^	Fish flour, cornmeal, soybean bran, wheat flour, corn gluten bran 60 *, rice grits, annatto extract, hydrolyzed poultry protein, autolyzed and dehydrated sugar cane yeast; vitamins: retinol acetate, monophosphate ascorbic acid (0.13%), cholecalciferol, tocopherol, menadione, thiamine, riboflavin, pyridoxine, cyanocobalamin, nicotinic acid, calcium pantothenate, folic acid, biotin, choline chloride, inositol; minerals: copper sulfate, iron sulfate, manganese monoxide, cobalt sulfate, calcium iodate, zinc sulfate, sodium selenite, sodium chloride, L-lysine, L-tryptophan, L-threonine, DL-methionine; yeast cell wall (0.3%), yucca extract, sodium and calcium aluminosilicate, propionic acid, acetic acid, formic acid, sorbic acid, butyl hydroxy anisole (BHA), butylhydroxytoluene (BHT), citric acid and ethoxyquin; dyes: tartrazine yellow, dry leaf green, and amaranth red.
Egg yolk powder + soybean oil ^3^	Pasteurized egg yolk powder and soybean oil in a ratio of 2.5:1 *w*/*v*.

Source: obtained from commercial labels. ^1^ The ingredient list information was obtained from [17]. ^2^ The ingredient list information was obtained from commercial labels (https://nutricon.ind.br/produtos/nutriflakes/). ^3^ The ingredient list information was obtained from the label of LIZA oil and Naturovos egg yolk (https://www.liza.com.br/produtos and https://www.tbca.net.br/base-dados/int_composicao_alimentos.php?cod_produto=BRC0009J). The ingredient lists were accessed on 10 October 2023. * Gene donor species: *Agrobacterium tumefacien*, *Bacillus thuringiensi*, *Streptomyces viridochromogene*.

**Table 2:** Centesimal composition of the diets offered to the control groups and obesity-induced groups.

Diets	*Artemia* sp. (100 g) ^1^	Commercial Fish Food (100 g) ^2^	Egg Yolk Powder + Soybean Oil (100 g) ^3^
Calories (Kcal)	391	291	737
Carbohydrates (g)	11	31 *	5
Proteins (g)	53	35	24
Lipids (g)	15	3	69

^1^ The information on the centesimal composition was obtained from [17]. ^2^ The information on the centesimal composition was obtained from commercial labels (https://nutricon.ind.br/produtos/nutriflakes/). ^3^ The information on the centesimal composition was obtained from the label of LIZA oil and Naturovos egg yolk (https://www.liza.com.br/produtos and https://www.tbca.net.br/base-dados/int_composicao_alimentos.php?cod_produto=BRC0009J). * Calculated by the difference between 100 and the sum of the percentages of water, protein, fat, minerals, and ash, according to the information on the label.

**Table 3:** Sequences of primers used for gene expression.

Gene	Forward Sequence (5’–3′)	Reverse Sequence (5’–3′)
*il-10*	TCACGTCATGAACGAGATCC	CCTCTTGCATTTCACCATATCC
*il-6*	AAG GGG TCA GGA TCA GCA C	GCT GTA GAT TCG CGT TAG ACA TC
*il-1β*	TGG CGA ACG TCA TCC AAG	GGA GCA CTG GGC GAC GCA TA
*tnf-α*	AAGGAGAGTTGCCTTTACCG	ATTGCCCTGGGTCTTATGG
*beta-actin*	CTGTTCCAGCCATCCTTCTT	TGTTGGCATACAGGTCCTTAC

Source: net primer—Primer Analysis site: https://www.idtdna.com/pages/tools/oligoanalyzer (accessed on 3 September 2023). *il-10*: Interleukin 10. Source: Sua et al. [26]; *il-6*: Interleukin 6; *il-1β*: Interleukin 1 beta; *tnf-α*: Tumor necrosis factor alpha. Source: Nuankaew et al. [27]. *Beta-actin*: Li et al. [28].

**Table 4:** Variation in dietary intake, caloric intake, and efficiency of zebrafish adults (three months) with diet-induced obesity (DIO) for eight weeks.

Groups Evaluated	∆ Dietary Consumption (g)	∆ Calorie Intake (KJ/Kcal Consumption)	∆ Caloric Efficiency (KJ/g of Weight)
Control 1	0.015	0.49	12.25
Control 2	0.004	0.13	1.02
Obesity 1	0.008	0.77	5.78
Obesity 2	0.060	1.97	12.07

## Data Availability

The original contributions presented in the study are included in the article.

## References

[1] World Obesity Atlas. https://www.worldobesity.org/resources/resource-library/world-obesity-atlas-2023.

[2] Pepe R.B., Lottenberg A.M.P., Fujiwara C.T., Beyruti M., Cintra D.E., Machado R.M., Rodrigues A., Jensen N.S., Caldas A., Fernandes A.E. Posicionamento Sobre o Tratamento Nutricional do Sobrepeso e da Obesidade Departamento de Nutrição da Associação Brasileira para o Estudo da Obesidade e da Síndrome Metabólica—ABESO—2022. https://abeso.org.br/wp-content/uploads/2022/11/posicionamento_2022-alterado-nov-22-1.pdf.

[3] Queiroz L.J., Medeiros I., Rosa S., Coimbra M., Camillo C.S., Paulo P., Guerra G.C.B., da Silva V.C., Schroeder H.T., Krause M. (2023). Efficacy of Carotenoid-Loaded Gelatin Nanoparticles in Reducing Plasma Cytokines and Adipocyte Hypertrophy in Wistar Rats. Int. J. Mol. Sci..

[4] Silva F.P., Araújo D., Carnier M., Paloma K.M., Valter T.B., Rischiteli A.B.S., Avila F., Pontes L.P.P., Hachul A.C.L., Neto N.I.P. (2021). Low dose of Juçara pulp (*Euterpe edulis Mart.*) minimizes the colon inflammatory milieu promoted by hypercaloric and hyperlipidic diet in mice. J. Funct. Foods..

[5] Laranjo L., Fortes N.C.L., Santiago H.C., Marcelo V.C., Maria A.G., Moura D. (2017). Obesity-induced diet leads to weight gain, systemic metabolic alterations, adipose tissue inflammation, hepatic steatosis, and oxidative stress in gerbils (*Meriones unguiculatus*). PeerJ.

[6] Sanders F.W.B., Acharjee A., Walker C., Marney L., Roberts L.D., Imamura F., Jenkins B., Case J., Ray S., Virtue S. (2018). Hepatic steatosis risk is partly driven by increased lipogenesis following carbohydrate consumption. Genome Biol..

[7] Saluja D., Jhanji R., Swati K., Bharti V., Neelam S., Rima S., Shelly A., Meena Y., Anoop K., Charan S. (2021). Importance of Zebrafish as an Efficient Research Model for the Screening of Novel Therapeutics in Neurological Disorders. CNS Neurol. Disord. Drug Targets.

[8] Dammski A., Müller B., Gaya C., Regonato D. (2011). Zebrafish Manual de Criação em Biotério. https://gia.org.br/portal/wp-content/uploads/2013/06/ZEBRAFISH.pdf.pdf.

[9] Zang L., Maddison A., Chen W. (2019). Zebrafish as a model for obesity and diabetes. Front. Cell Dev. Biol..

[10] Adhish A., Manjubala I. (2023). Effectiveness of zebrafish models in understanding human diseases—A review of models. Heliyon.

[11] Howe K., Clark M.D., Torroja C.F., Torrance J., Berthelot C., Muffato M., Collins J.E., Humphray S., McLaren K., Matthews L. (2013). The zebrafish reference genome sequence and its relationship to the human genome. Nature.

[12] Montalbano G., Mania M., Abbate F., Navarra M., Guerrera M.C., Laura R., Vega J.A., Levanti M., Germanà A. (2018). Melatonin treatment suppresses appetite genes and improves adipose tissue plasticity in diet-induced obese zebrafish. Endocrine.

[13] Meguro S., Hasumura T., Hase T. (2015). Body Fat Accumulation in Zebrafish Is Induced by a Diet Rich in Fat and Reduced by Supplementation with Green Tea Extract. PLoS ONE..

[14] Oka T., Nishimura Y., Zang L., Hirano M., Shimada Y., Wang Z., Umemoto N., Kuroyanagi J., Nishimura N., Tanaka T. (2010). Diet-induced obesity in zebrafish shares common pathophysiological pathways with mammalian obesity. BMC Physiol..

[15] Westerfield M. (2007). O Livro do Peixe-Zebra, 5ª Edição; Um Guia Para o uso Laboratorial de Peixe-Zebra (Danio Rerio).

[16] Landgraf K., Schuster S., Meusel A., Garten A., Riemer T., Schleinitz D., Kiess W., Körner A. (2017). Short-term overfeeding of zebrafish with normal or high-fat diet as a model for the development of metabolically healthy versus unhealthy obesity. BMC Physiol..

[17] Jamali H., Ahmadifard N., Noori F., Agh N., Gisbert E. (2018). Improving coofeeding strategies for Neotropical green terror cichlid (*Aequidens rivulatus*) larvae with lecithin-enriched Artemia franciscana nauplii: Effects on survival, growth performance and body composition. Aquac. Res..

[18] UNIFESP Universidade Federal de São Paulo. Comissão de Ética no Uso de Animais. https://site.unifesp.br/ceua/images/documentos/CEUA/Guia_Eutanasia_CEUA_UNIFESP_2024.pdf.

[19] Barcellos L.J.G., Ritter F., Kreutz L.C., Quevedo R.M., Silva L.B., Bedin A.C., Finco J., Cericato L. (2007). Whole-body cortisol increases after direct and visual contact with a predator in zebrafish, Danio rerio. Aquac. Res..

[20] Vendrame S., Daugherty A., Kristo A.S., Riso P., Klimis-Zacas D. (2013). Wild blueberry (*Vaccinium angustifolium*) consumption improves inflammatory status in the obese Zucker rat model of the metabolic syndrome. J. Nutr. Biochem..

[21] Martins L.B., Oliveira M.C., Menezes-Garcia Z., Rodrigues D.F., Lana J.P., Vieira L.Q., Teixeira M.M., Ferreira A.V.M. (2017). Paradoxical role of Tumor of Necrosis Factor on metabolic dysfunction and adipose tissue expansion in mice. Nutr. J..

[22] Luz A.B.S., Figueredo J.B.S., Salviano B.D.P.D., Aguiar A.J.F.C., Pinheiro L.G.S.D.P., Camillo C.S., Maciel B.L.L., Morais A.H.A. (2018). Adipocytes and intestinal epithelium dysfunctionslinking obesity to inflammation induced by High Glycemic Index Pellet-diet in Wistar. Biosci. Rep..

[23] Martins F.F., Marinho T.S., Cardoso L.E.M., Silva S.B., Mello V.S., Aguila M.B., Lacerda C.A.M. (2022). Semaglutide (GLP-1 receptor agonist) stimulates browning on subcutaneous fat adipocytes and mitigates inflammation and endoplasmic reticulum stress in visceral fat adipocytes of obese mice. Cell Biochem. Funct..

[24] Tschanz S.A., Burri P.H., Weibel E.R. (2011). A simple tool for stereological assessment of digital images: The STEPanizer. J. Microsc..

[25] Bustin S.A., Benes V., Garson J.A., Hellemans J., Huggett J., Kubista M., Mueller R., Nolan T., Pfaffl M.W., Shipley G.L. (2009). The MIQE Guidelines: Minimum Information for Publication of Quantitative Real-Time PCR Experiments. Clin. Chem..

[26] Sua B.C., Laia Y.W., Chena J.Y., Yu C. (2018). PanTransgenic expression of tilapia piscidin 3 (TP3) in zebrafish confers resistance to Streptococcus agalactiae. Fish Shellfish. Immunol..

[27] Nuankaew W., Lee H.K., Nam Y.H., Shim J.H., Kim N.W., Shin S.W., Kim M.C., Shin S.Y., Hong B.N., Dej-adisai S. (2022). The Effects of Persimmon (Diospyros kaki L.f.) Oligosaccharides on Features of the Metabolic Syndrome in Zebrafish. Nutrients.

[28] Li P., Li Z.-H., Zhong L. (2019). Effects of low concentrations of triphenyltin on neurobehavior and the thyroid endocrine system in zebrafish. Ecotoxicol. Environ. Saf..

[29] Moreira A.L.P., Luchiari A.C. (2022). Effects of oxybenzone on zebrafish behavior and cognition. Sci. Total Environ..

[30] Jin Y., Kozan D., Anderson J.L., Hensley M., Shen M.C., Wen J., Moll T., Kozan H., Rawls J.F., Farber S.A. (2023). A high-cholesterol zebrafish diet promotes hypercholesterolemia and fasting-associated liver triglycerides accumulation. bioRxiv.

[31] Li L., Chen J., Sun H., Niu Q., Zhao Y., Yang X., Sun Q. (2023). Orm2 Deficiency Aggravates High-Fat Diet-Induced Obesity through Gut Microbial Dysbiosis and Intestinal Inflammation. Mol. Nutr. Food Res..

[32] Teixeira B.C., Lopes A.L., Macedo R.C.O., Correa C.S., Ramis T.R., Ribeiro J.L., Reischak-Oliveira A. (2014). Inflammatory markers, endothelial function and cardiovascular risk. J. Vasc. Bras..

[33] Ang Z., Ding J.L. (2016). GPR41 and GPR43 in Obesity and Inflammation—Protective or Causative?. Front. Immunol..

[34] Banerjee D., Patra D., Sinha A., Roy S., Pant R., Sarmah R., Dutta R., Bhagabati S.K., Tikoo K., Pal D. (2022). Lipid-induced monokine cyclophilin-A promotes adipose tissue dysfunction implementing insulin resistance and type 2 diabetes in zebrafish and mice models of obesity. Cell. Mol. Life Sci..

[35] Nielsen K.N., Peics J., Ma T., Karavaeva I., Dall M., Chubanava S., Basse A.L., Dmytriyeva O., Treebak J.T., Gerhart-Hines Z. (2018). NAMPT-mediated NAD+ biosynthesis is indispensable for adipose tissue plasticity and development of obesity. Mol. Metab..

[36] Lempesis I.G., Meijel R.L.J., Manolopoulos K.N., Goossens G.H. (2019). Oxygenation of adipose tissue: A human perspective. Acta Physiol..

[37] Minchin J., Rawls J.F. (2017). A classification system for zebrafish adipose tissues. Dis. Model. Mech..

[38] Türkoğlu M., Baran A., Sulukan E., Ghosigharehagaji A., Yildirim S., Ceyhun H.A., Bolat I., Arslan M., Ceyhun S.B. (2021). The potential effect mechanism of high-fat and high-carbohydrate diet-induced obesity on anxiety and offspring of zebrafish. Eating and Weight Disorders—Studies on Anorexia. Bulim. Obes..

[39] Alonso-Caraballo Y., Hodgson K.J., Morgan S.A., Ferrario C.R., Vollbrecht P.J. (2019). Enhanced anxiety-like behavior emerges with weight gain in male and female obesity-susceptible rats. Behav. Brain Res..

[40] Azbazdar Y., Poyraz Y.K., Ozalp O., Nazli D., Ipekgil D., Cucun G., Ozhan G. (2023). High-fat diet feeding triggers a regenerative response in the adult zebrafish brain. Mol. Neurobiol..

[41] Yehuda H., Madrer N., Goldberg D., Soreq H., Meerson A. (2023). Inversely Regulated Inflammation-Related Processes Mediate Anxiety-Obesity Links in Zebrafish Larvae and Adults. Cells.

[42] Banks W.A. (2019). The blood–brain barrier as an endocrine tissue. Nat. Rev. Endocrinol..

[43] Lima R.R., Costa A.M.R., Souza R.D., Gomes-Leal W. (2007). Inflamação em doenças neurodegenerativas. Rev. Para Med..

[44] Berlanga-Acosta J., Guillén-Nieto G., Rodríguez-Rodríguez N., Bringas-Veja M.L., García-del-Barco-Herrera D., Berlanga-Saez J.O., García-Ojalvo A., Valdés-Sosa M.J., Valdés-Sosa P.A. (2020). Insulin Resistance at the Crossroad of Alzheimer Disease Pathology: A Review. Front. Endocrinol..

